# Which panic-agoraphobic symptoms could be associated with the presence of autistic traits among patients with panic disorder?

**DOI:** 10.1017/S1092852925000240

**Published:** 2025-04-10

**Authors:** Barbara Carpita, Chiara Bonelli, Francesca Parri, Gianluca Cerofolini, Cristiana Pronestì, Benedetta Nardi, Diego Laureti, Gabriele Massimetti, Ivan Mirko Cremone, Stefano Pini, Andrea Fiorillo, Liliana Dell'Osso

**Affiliations:** 1 Department of Clinical and Experimental Medicine, University of Pisa, Pisa, Italy; 2 Department of Biotechnology, Chemistry and Pharmacy, University of Siena, Siena, Italy; 3 Department of Psychiatry, University of Campania “Luigi Vanvitelli”, Naples, Italy

**Keywords:** autism spectrum disorder, autistic traits, panic disorder, agoraphobic disorder, panic-agoraphobic symptoms

## Abstract

**Objective:**

Panic disorder (PD) is a psychopathological condition characterized by short-lived anxiety attacks in association with several physical symptoms, followed by the fear of other attacks’ possible occurrence or their consequences and avoidance behaviors. Recent literature highlighted a significant correlation between autism spectrum disorder (ASD) and anxiety disorder. To date, the specific association between PD and autistic traits (ATs) remains to be clarified. This study aimed to investigate the presence of an autism spectrum in patients with PD, specifically focusing on the presence of possible factors associated with these conditions.

**Methods:**

The study recruited 112 subjects: 55 subjects with a clinical diagnosis of PD and 57 healthy controls (HCs). All participants were evaluated with the Panic Agoraphobic Spectrum—Short Version (PAS-SV) and the Adult Autism Subthreshold Spectrum (AdAS Spectrum) questionnaire.

**Results:**

Results outlined a significant correlation between agoraphobic-panic symptoms and autism spectrum, with significantly positive correlations of all AdAS Spectrum domain scores, as well as its total score with all PAS-SV domains as well as with its total score. Moreover, atypical panic symptoms, and anxious expectation, and maladaptive behaviors were factors associated with a possible underlying presence of higher AT in PD subjects.

**Conclusions:**

Our findings support the association between the panic-agoraphobic dimension and ASD. Indeed, increased reactions to stressful life events due to autistic altered sensory reactivity may increase fears responses such as anxious manifestations and maladaptive behaviors.

## Introduction

Panic disorder (PD) is a psychopathological condition included in the section “Anxiety Disorders” of the Diagnostic and Statistical Manual of Mental Disorders, 5th edition, text revised (DSM-5 TR).^1^ The clinical picture is characterized by short-lived anxiety attacks reaching their peak intensity in a few minutes in association with several physical symptoms.^1^ Moreover, panic attacks are frequently followed by the fear of other attacks’ possible occurrence or their consequences and avoidance behaviors, consisting of several maladaptive strategies to avoid the onset of a new crisis.^1^ To date, PD occurs together with agoraphobic disorder (AP) with a lifetime prevalence ranging between 0.5% and 1.8%.^2^^,^^3^ Indeed, PD often shares comorbidity with other anxiety disorders (AD), major depression, and bipolar disorder.^1^

A significant correlation between autism spectrum disorder (ASD) and AD has been recently highlighted.^4^^,^^5^ Noteworthy, AD range around 40% in young ASD patients,^5^ while in adults they reach 84%.^4^ Prevalence of AD in autistic subjects is at least five times higher than in the general population.^6^ For this reason, mood and anxious symptoms may overlap with ASD, making the diagnostic process more complex.^7^ Recent research identified alexithymia, emotion regulation deficits, unknown intolerance, and sensory processing alterations as some of the most significant factors contributing to the development of anxious symptoms, and more specifically panic-agoraphobic symptoms, in autism.^8^^,^^9^

According to the dimensional approach of the “Spectrum Model”,^10^^–^^13^ symptoms related to mental disorders are conceptualized as a continuum from the general to the clinical population, highlighting the importance of investigating not only full-fledged clinical pictures but also those symptoms and traits presenting as isolated, atypical, or subthreshold features, which do not reach the diagnostic criteria but may nevertheless exert an impact on quality of life and/or complicate other comorbid conditions.^10^^–^^13^

In this framework, an increasing body of studies is highlighting the role of subthreshold autism symptoms in several psychiatric disorders.^14^ The presence of a subthreshold autism spectrum was firstly investigated among first-degree relatives of ASD probands, who often reported personal characteristics, behavioral and cognitive traits qualitatively similar to ASD symptoms, although not sufficient in number and/or intensity for reaching the clinical diagnosis.^15^ Subsequently, autism spectrum symptoms were progressively conceptualized as a dimension continuously distributed in different populations, with complete neurotypicality and full-fledged ASD at the extreme ends.^14^

Moreover, autistic traits (ATs) seem particularly represented in specific populations, such as psychiatric patients affected by other mental conditions, for which ATs seem to constitute a vulnerability factor and contemporarily playing a detrimental role in the course of the disorder, including implying a higher suicidality risk.^16^^–^^19^

Despite several studies examined the existing relationship between anxiety disorder and ASD, and another study of our group highlighted greater panic-agoraphobic symptoms among patients with ASD^20^ to date the specific association between PD and ATs remains to be clarified. In this perspective, this study aimed to investigate the presence of an autism spectrum in patients with PD, specifically focusing on the presence of possible factors associated with these conditions.

## Materials and methods

### Study sample and procedures

For this study, a total sample of 112 subjects was recruited between September 2022 and March 2023 at the Psychiatric clinic of the University of Pisa: 55 subjects with a clinical diagnosis of PD and 57 healthy controls (HCs). Subjects with intellectual disability, poor collaboration skills, an age under 18, language or intellectual impairment that would make it difficult to complete the exams, and continuous psychotic symptoms were excluded from the recruitment procedures. The study was conducted in accordance with the Declaration of Helsinki, and the local Ethical Committee approved the study protocol. All participants provided a written informed consent following a thorough explanation of the study and a chance for questions. All participants were evaluated with the Panic Agoraphobic Spectrum—Short Version (PAS-SV) and the Adult Autism Subthreshold Spectrum (AdAS Spectrum) questionnaire.

### Measures

#### The PAS-SV

The PAS-SV is a self-report questionnaire composed of 78 dichotomous items organized in 4 domains: panic symptoms, atypical panic symptoms, anxious expectation and maladaptive behavior, and agoraphobia. The purpose of the questionnaire was to provide a valuable instrument for clinical practice and the study of panic-agoraphobic tendencies and panic attacks, both in their full-blown and milder subthreshold forms. During the validation study, the questionnaire showed good psychometric properties. **^21^**

#### The AdAS spectrum

The AdAS Spectrum questionnaire is a 160-item self-report measure used to assess a wide variety of autism-related symptoms in individuals without cognitive or language impairments. The questionnaire is organized in 7 domains: *childhood and adolescence*, *verbal* and *nonverbal communication*, *empathy*, *inflexibility and adherence to routine*, *restricted interests and rumination*, and *hyper- and hyporeactivity to sensory input.* The questionnaire demonstrated strong internal consistency, outstanding test–retest reliability, and convergent validity with other dimensional measures of the autism spectrum were seen in the validation research. **^22^** The questionnaire also has two validated cut-off scores: 70 for identifying subjects with full-blown ASD and 43 for determining the presence of significant AT. **^23^**

### Statistical analysis

Chi-square and *t*-Student tests were used to compare sociodemographic characteristics between the two diagnostic groups. A further Chi-square analysis was used to compare the prevalence of clinically relevant ATs or symptoms of possible full-blown ASD, based on the threshold score of the AdAS Spectrum, between the two diagnostic groups.

Subsequently, t-Student tests were used to compare AdAS Spectrum total and domain scores between the two groups. Pearson’s correlation coefficients were then used to investigate the presence of significant correlations between AdAS Spectrum’s and PAS-SV domain scores.

Lastly, using the AdAS Spectrum total score as the dependent variable and the PAS-SV domain scores as the independent variables, a linear regression analysis was performed in order to investigate the presence of possible factors associated with higher AdAS Spectrum total scores among PAS-SV domains. All statistical analyses were performed with SPSS version 26.0.

## Results

The total sample was made of 112 subjects divided in two categories: 55 subjects belonged to the PD (F=36; M=19; mean age: 42.22 ± 12.41 years) group and 57 to the HCs group (F=31; M=26; mean age: 38.21 ± 13.14 years). The two groups did not significantly differ for age or gender (see Table 1). Chi-square analysis showed that the PA group had a significantly higher percentage of subjects that showed clinically relevant ATs and full-blown ASD, according to the AdAS Spectrum threshold scores, compared to HCs (see Table 2). Student *t*-test results highlighted how PD subjects scored significantly higher than HCs in all AdAS Spectrum domains as well as in its total score (see Table 3). According to Pearson correlation analysis, all AdAS Spectrum domain scores as well as its total, were significantly positively correlated with all PAS-SV domains, as well as with its total (see Table 4). All correlations ranged from moderate to strong, with the only exception of the PAS-SV *Agoraphobia* domain and the AdAS Spectrum *Empathy* domain, for which the correlation coefficient was weak. Higher correlation coefficient scores were reported between the AdAS Spectrum total score and the PAS-SV total and domain scores. From the linear regression analysis conducted, for which the AdAS Spectrum total score was used as the dependent variable and PAS-SV domain scores as independent variables, the PAS-SV *Atypical panic symptoms* and *Anxious expectation and maladaptive behaviors* domains emerged as significant factors associated with a higher AdAS Spectrum total score (see Table 5).

**Table 1.** Age and Gender Comparison between Diagnostic Groups

		PD	HCs	*t*	*p*
		*Mean ± SD*	*Mean ± SD*		
Age		42.22 ± 12.41	38.21 ± 13.14	1.658	.100
		*n*(%)	*n*(%)	Chi-square	*p*
Gender	F	36 (65.5%)	31 (54.4%)	1.427	.232
	M	19 (34.5%)	26 (45.6%)

**Table 2.** Comparison of Frequency of Clinically Relevant ATs or Full-Blown ASD in the Two Diagnostic Groups

	No ASD*n*(%)	ATs*n*(%)	ASD*n*(%)	Chi-square	*p*
PA	33 (60.0%)	19(34.5%)	3(5.5%)	28.373	<.001
HC	57(100.0%)	0(0.0%)	0(0.0%)		

Student *t*-test results reported in Table 3 highlighted how PD subjects scored significantly higher than HCs in all AdAS Spectrum domains as well as in its total score (see Table 3).

**Table 3.** Comparison of Scores Reported on AdAS Spectrum between Groups

	HC*Mean ± SD*	PD*Mean ± SD*	*t*	*P*
Child/adolesc.	0.67 ± 1.11	6.69 ± 5.91	7.432	<.001*
Verbal comm.	0.47 ± 0.60	3.76 ± 3.33	7.220	<.001*
Nonverbal comm.	0.98 ± 1.17	6.27 ± 5.87	6.562	<.001*
Empathy	0.19 ± 0.48	2.22 ± 2.77	5.339	<.001*
Inflex. and adher. to routine	1.49 ± 1.67	8.22 ± 5.45	8.757	<.001*
Restricted interests and rumination	0.86 ± 1.33	5.84 ± 4.98	7.168	<.001*
Hyper/hyporeact. to sensory inputs	0.31 ± 0.57	4.62±4.36	7.257	<.001*
Total score	4.98 ± 4.24	37.62 ± 19.52	12.127	<.001*

* *p<.001*

**Table 4.** Correlations between AdAS Spectrum and PAS-SV Domains and Total Scores in the Overall Sample

	Panic sympt.	Atypical sympt.	Anx. Exp./Malad. behav.	Agoraph.	Panic sympt. percept.	Tot. PAS-SV
Child/adolesc.	.533**	.576**	.476**	.480**	.533**	.613**
Verbal comm.	.528**	.545**	.525**	.370**	.528**	.534**
Nonverbal comm.	.480**	.516**	.489**	.357**	.480**	.500**
Empathy	.507**	.507**	.411**	.255**	.507**	.451**
Inflex. and adher. to routine	.488**	.420**	.554**	.505**	.488**	.538**
Restric. interests/ rumination	.450**	.500**	.605**	.442**	.450**	.550**
Hyper/hyporeact. to sensory inputs	.577**	.660**	.620**	.408**	.577**	.616**
Total score	.672**	.701**	.746**	.557**	.672**	.730**

** *p<.001*

**Table 5.** Linear Regression Analysis with PAS-SV Domain Scores as Independent Variables and AdAS Spectrum Total Score as the Dependent Variable

	*b* (SE)	BETA	*t*	*p*
*Constant*	6.032 (2.040)		2.957	.004*
Panic symptoms	−1.258(.889)	−.281	−1.416	.160
Atypical panic symptoms	1.883(.792)	.425	2.377	.019*
Anx. Expect./ Maladap. behav.	2.101(.488)	.675	4.308	<.001*
Agoraphobia	−.230(.445)	−.054	−.516	.607

*R*^2^: .583; adjusted *R*^2^: .568.

* *p<.001*

## Discussion

This study highlighted a significant correlation between agoraphobic-panic symptoms and autism spectrum. According to our data, patients with PD reported higher scores at AdAS Spectrum questionnaire. Conversely, all AdAS spectrum domain scores, and its total were positively correlated with all PAS-SV domains and with the PAS-SV total score.

Research reported dysfunctional strategies in cognitive emotion regulation as a risk factor for AD.^24^ Autistic subjects, in turn, are more prone since childhood to negative expectations about the future, poorer beliefs about their abilities, and greater self-blame. These dysfunctional cognitive evaluations are associated with increased levels of anxiety^25^^,^^26^ and maladaptive behaviors^27^^–^^29^ related to PD. Current literature revealed a strong association between ASD and AD, although underlying psychopathological mechanisms remain poorly understood.^30^^–^^33^ To date, very few studies have focused on the presence of ATs in subjects with PD without a formal diagnosis of ASD.^20^ Panic-agoraphobic symptoms are a transdiagnostic entity often associated with a wide range of atypical and subthreshold manifestations not necessarily codified in the DSM criteria. Among these, maladaptive behaviors such as increased sensitivity to medication and reassurance, and separation anxiety are also present in several patients suffering from PD or AP.^11^ In this perspective, several studies widely demonstrated that ASD subjects may express anxiety throughout atypical patterns or maladaptive behaviors, including unusual phobias.^34^^,^^35^ According to our findings, both the specific domains of PAS-SV “atypical symptoms” and “anxious expectation/maladaptive behaviors” showed the strongest associations with the “Hyper/Hypo Reactivity to sensory inputs” of AdAS Spectrum questionnaire. Previous research highlighted a significant correlation between autistic atypical responses to sensory inputs, and the atypical anxious manifestations or maladaptive behaviours.^34^^,^^36^^,^^37^ In this framework, sensory processing difficulties may predict anxiety manifestations in ASD subjects.^37^^–^^44^ Finally, a tangible connection between the altered perceptions processing and emotional problems was observed in young ASD patients using electrophysiological studies.^45^

Our study also showed that PD subjects who scored higher at the *Hyper/hyporeactivity to sensory inputs* domain of the AdAS spectrum also presented higher levels of “Panic symptoms” and “Panic symptoms perception” domain scores. Starting from a neuro-anatomic point of view, this relationship could be explained by the role of the altered reactivity to sensory stimuli^46^ due to limbic and glutamatergic system alterations.^46^^–^^48^ According to the “Intense World Theory,” autistic subjects present hyperactivation of the glutamatergic circuits, leading to hyper-perception, hyper-attention, and hyper-memory, together with hyper-emotionality deriving from amygdala altered function.^49^^,^^50^ Noteworthy, when present at extreme levels, the perception of external stimuli may become extremely intense, and the memory of stressful events will also be more persistent and vivid, making these subjects unable to manage the external world information.^49^^–^^51^ In this perspective, the re-experiencing of minor traumatic events, due to the intense autistic ruminative activity, leads to the development of post-traumatic stress trajectories.^20^ As a result, autistic subjects would manifest increased reactions to stressful life events.^31^^,^^49^^,^^52^^–^^55^ Therefore, minimal stressing stimuli could represent a significant trigger to determine exaggerated fear responses such as anxious manifestations and maladaptive behaviors.^49^ Moreover, while the autistic-like atypical brain may increase the ability to perform certain tasks in which the autistic subject excels, on the other hand, it could favor the development of repetitive behaviors and adherence to routines in order to reduce the possibility of encountering adverse or unexpected events.^56^ These behaviors, to a certain extent, may recall the maladaptive behaviors that patients with PD put into practice, with the purpose of reducing the risk of adverse events that could trigger a panic attack. Thus, our findings confirm previous literature. Indeed, linear regression analyses revealed that “atypical panic symptoms” and “anxious expectation and maladaptive behaviors” PAS-SV domains are factors associated with a higher AdAS Spectrum total score.

Noteworthy, *anxious expectation and maladaptive behavior* the PAS-SV domain was also highly correlated with the AdAS “Restricted Interests and Rumination” domain. Recurrent thinking is related to panic attacks and fear of consequences^52^^,^^57^ and may predict PD or AP.^58^^,^^59^ However, ruminative thinking recurs in several psychiatric conditions, such as ASD,^60^^,^^61^ hampering the correct copying strategies through the re-experiencing of stressful life events.^57^^,^^62^ The restricted and repetitive behaviors/thinking would represent an attempt to adapt to stressful external stimuli, overrepresented and altered in the world of autistic subjects,^49^ thus increasing anxiety. Moreover, the scarce tolerance to uncertain situations and the consequently need to maintain rigid patterns of thinking and behavior would justify increased anxious manifestations in autistic subjects.^63^^–^^65^ In line with this model, several domains of PAS-SV were positively related to “Inflexibility and Adherence to routine” domain. According to our findings, autistic impairments in verbal (and nonverbal) communication could also contribute to the correlation between AT and several PAS-SV domains. Indeed, altered communication leads to a reduced verbalization of internal stressful thinking and feelings, with consequent anxious manifestations, including both typical and atypical anxious symptoms.^35^^,^^66^^,^^67^ Furthermore, it is known that the limited skills in social interactions, as well as the misunderstanding of social signals^68^^,^^69^ expose autistic subjects to social rejection, embarrassment, and bullying.^70^^,^^71^ These episodes may lead to the increase of anxiety, anxious expectations, and avoidance of social events, with the occurrence of panic-agoraphobic symptoms.

This study should be considered in light of some limitations. First of all, the small sample size may limit the generalizability of our results. Moreover, the use of self-reported instruments may expose to bias related to over- or under-estimation of symptoms by participants. In addition, the cross-sectional design of the study did not allow us to make inferences about possible temporal or causal relationships between the investigated variables. Finally, our work has been conducted on a non-autistic population, and results might be not generalizable to autistic subjects.

Globally, our study confirmed the existence of a statistically significant relationship between autism and panic-agoraphobic spectra. PD subjects reported higher AT, with all AdAS Spectrum domain scores as well as its total significantly positively correlated with all PAS-SV scores. Results highlighted that *atypical panic symptoms*, and *anxious expectations, and maladaptive behaviors* are factors associated with an underlying presence of higher AT in PD subjects. In this framework, autistic altered reactivity to sensory input may play a central role. Increased reactions to stressful life events may lead to increased fear responses, such as anxious manifestations and maladaptive behaviors ascribable to PD dimensions.

## Data Availability

All data generated or analyzed during this study are included in this published article.
